# Brine Baths for Rheumatism

**Published:** 1888-11-03

**Authors:** 


### Brine Baths for Rheumatism.

By a Sufferer.

A bad attack of inflammatory rheumatism, which settled in the joints and threatened to become chronic, was the occasion for much well-intentioned and gratuitous advice being showered on the sufferer. For instance, attention was besought to St. Jacob's Oil, Elliman's Embrocation, and various other nostrums which had either given relief in certain known cases, or were reported to have done so. Then sulphur rubbed in the stockings and a horrible mixture of the same with spirits taken fasting was recommended as an infallible cure; a hare's foot fastened to the affected joints and worn day and night, or a *stolen* (in that lay the charm) potato, cut up and cherished in the same localities, would be certain to relieve. Time, however, would fail to recapitulate the hundred and one "sure and safe cures" heard with faithless ears and sinking heart by the sufferer, who doubtfully tried some of the things recommended, but with slight or no effect. His was either a very stubborn case, or more faith was required to ensure success, probably a mixture of both, since the faculty, alas! took a gloomy view of the situation, pronounced the attack to be Rheumatroid Arthritis, and prescribed simply constant change of air and scene, accompanied by a liberal diet. So the patient drifted aimlessly about from one place to another, sometimes gaining strength, but more often losing it, the progression to health being of the retrograde order. At length, two very severe winters caused such prostration of strength that it was decided a supreme effort must be made, and some baths tried. Aix-les-Bains, Wiesbaden, Kreutznach were recommended by sympathising friends, and when declined on the score of distance and the trouble and fatigue of reaching them, Buxton, Woodhall Spa, and Droitwich were substituted. The difficulty of making a choice and a natural inertness now intervened. At this juncture a "being" appeared in the flesh who six or seven years before had really been cured at Droitwich. Seeing is believing, and the sufferer felt here was something tangible, and that no time should be lost in seeking to gain a like benefit. A slightly rheumatic friend was pressed into the service as "compagnon de voyage," apartments taken, and in the midst of the Jubilee rejoicings the two found themselves settled at Droitwich. A few words about this tumble-down and rather dirty place may not here be inappropriate. From the railway the town does not present a very inviting appearance, and the traveller is unfavourably impressed by a drive through the High Street, which presents the appearance of a crescent with the horns standing upwards, though within the last twenty years it was perfectly level. The immense quantities of brine drawn out by the salt works has caused the earth to subside, and other parts of the town also show signs of an impending collapse, shutters and doors hanging aimlessly on their hinges, many of the former refusing to close at all, and the latter having openings between them and the sides highly favourable to ventilation. A wall on the Worcester Road, which ten years since was ten feet high, of substantial build, with fine coped top, has disappeared in the earth, the rounded bricks alone showing where it once stood. Factory chimneys and church towers aim at rivalling the Leaning Tower of Pisa, and St. Andrew's Church itself is so crippled by subsidence and the action of the brine-laden air, which causes the stone to flake off, that it has been closed for several years. Subscriptions, are, however, now being raised to render it fit for use, and, metaphorically speaking, set it on its legs. But, as compensation for all these drawbacks, the air is wonderfully salubrious, epidemics never visit Droitwich, fever and diphtheria are almost unknown, and not a solitary case of cholera occurred in any of the fearful visitations which, in 1834 and since, have scourged Great Britain. The apparently unstable condition of the town is necessarily alarming to a stranger, but the inhabitants live at ease, "careless and secure," and the visitor, profiting by their example, soon learns to dismiss any forebodings from his mind, and to feel the place will probably last his time.

Our sufferer, having also arrived at this philosophical conclusion by a severe mental regimen of Marcus Aurelius, cast about for some means of conveyance to the Brine Baths, and found there were two bath chairs for the accommodation of invalids, one of which was immediately secured for daily use.

As soon as the sufferer recovered from his journey, he proceeded to the Royal Brine Baths—a modest building situated at the back, and in the grounds of, the Royal Hotel. The establishment consists of first, second, and third class hot baths, and a large cold swimming bath all under the same roof. A waiting-room, where tickets are procured, is on the opposite side of the enclosure, which in the baking sun of 1887 presented a desolate appearance, a little mangy grass-plot and a few dried-up shrubs being its only embellishment.

Inside, the accommodation is good, each bath having two dressing-rooms, as at Buxton, thus diminishing for the bathers the tedium of waiting. The baths themselves are made of wood, the action of the water quickly wearing away stone, tiles, or enamel. Our sufferer disrobed in a dressing-room, in which was a comfortable little couch, and after shuffling, with the aid of the attendant, the few necessary steps to gain the bath, was deposited in a mixture of hot salt water and pure cold brine at a temperature of 98 deg. Fahr., and was immediately seized by an irresistible desire to turn on his face heels upwards, the brine being more buoyant than any sea water round the coast of England; but he was soon righted, and by the aid of a wooden bar fixed across the bath shortly learned to float face upwards, and so enjoyed the soothing sensation caused by the hot liquid in which he was immersed.

Here he remained twenty minutes (timed by an hour glass) during which the attendant came in frequently to see if all were right, to keep up the heat of the water, and to use the flesh brush if desired.

At the expiration of the twenty minutes the bather rang a bell, the rope of which was in close proximity, and the attendant brought two large hot Turkish sheets, wherewith the patient was enveloped to prevent a chill on emerging from the extreme heat of the water, and was speedily deposited on the couch in his dressing-room, there to remain a quarter of an hour, perspiring copiously if possible, as it carries off the acidity from the blood; then, cooling gently, he dressed and returned to his apartments. This treatment was adhered to daily for several weeks, with the effect of ameliorating the complaint, but causing much feebleness and exhaustion. In consequence of this our sufferer then removed to Malvern, where the pure bright air and cheerful surroundings of that lovely spot completed the cure, thus confirming the general experience that the full benefit of the treatment is not realised during the course itself.

The convalescent learns that this year, 1888, the inhabitants of Droitwich have apparently awoke to the desirability of making their town more attractive and wider known. Paint and whitewash have been liberally used, and advertisements of the New St. Andrew's Baths appear at many railway stations. By these exertions it is hoped that this quaint little health resort will soon be fully and deservedly appreciated.

